# A Case of Longstanding Idiopathic Pernio/Chilblain Disease

**DOI:** 10.7759/cureus.17674

**Published:** 2021-09-03

**Authors:** Diva C Maraj, Ronda Barak-Norris

**Affiliations:** 1 Allergy and Immunology, St. Joseph Mercy Oakland Hospital, Pontiac, USA; 2 Allergy and Immunology, Beaumont Hospital, Royal Oak, USA; 3 Allergy and Immunology, Ascension Providence Hospital, Southfield, USA

**Keywords:** pernio, chilblain's disease, rheumatology, skin, blisters

## Abstract

Pernio, also known as chilblains, presents as erythematous macules at sites of cold exposure, mainly in women. It is a diagnosis that is often overlooked, and when suspecting a patient with pernio, other conditions such as lupus nephritis and Raynaud’s must be ruled out. A 46-year-old lady presented to the clinic with skin findings suggestive of pernio. She had erythematous macules on the dorsum of her hands, which appeared during cold weather and lasted for three weeks. She had been suffering with this condition for over 18 years and nothing has helped her condition, other than preventing cold exposure. There are limited treatment options for pernio, and current management includes using steroids, calcium-channel blockers and cold avoidance. Current research has suggested that pernio could also be linked to the severe acute respiratory syndrome coronavirus 2.

## Introduction

Pernio, also known as chilblains, presents as single or multiple erythematous to violaceous macules, papules, plaques, or nodules at sites of cold exposure and commonly occurs in the fingers and toes [1]. Symptoms usually develop 12-24 hours after cold exposure, with temperatures around 12-15°C and tend to resolve within one to three weeks [1]. Lesions may also blister and ulcerate and there is a poorly understood pathophysiology of the disease; however, it seems to be caused by vasospasm of the vessels [2]. There have also been numerous studies showing a possible connection between pernio and the severe acute respiratory syndrome coronavirus 2 (SARS-CoV-2); however, no concrete relationship has been established thus far [3]. Pernio mainly affects young and middle-aged women, usually before the age of 40, with a prevalence of 0.9-2.1 per 1000 in women and 0.6 per 1000 in men [4]. Risk factors for pernio include female gender, cold weather, a low BMI and smoking [4]. Pernio is often found in colder climates, such as in the UK; however, it can also be seen worldwide [5]. Pernio is a diagnosis that is often overlooked or missed resulting in patients experiencing unnecessary testing and insufficient management of the condition. A large regressional study found that chilblain-like eruptions were a prognostic factor for coronavirus disease 2019 (COVID-19); however, it seemed to be a protective response against COVID-19 and appeared in warm weathers, as opposed to the usual cold weather that triggers pernio [5].

## Case presentation

**Figure 1:**
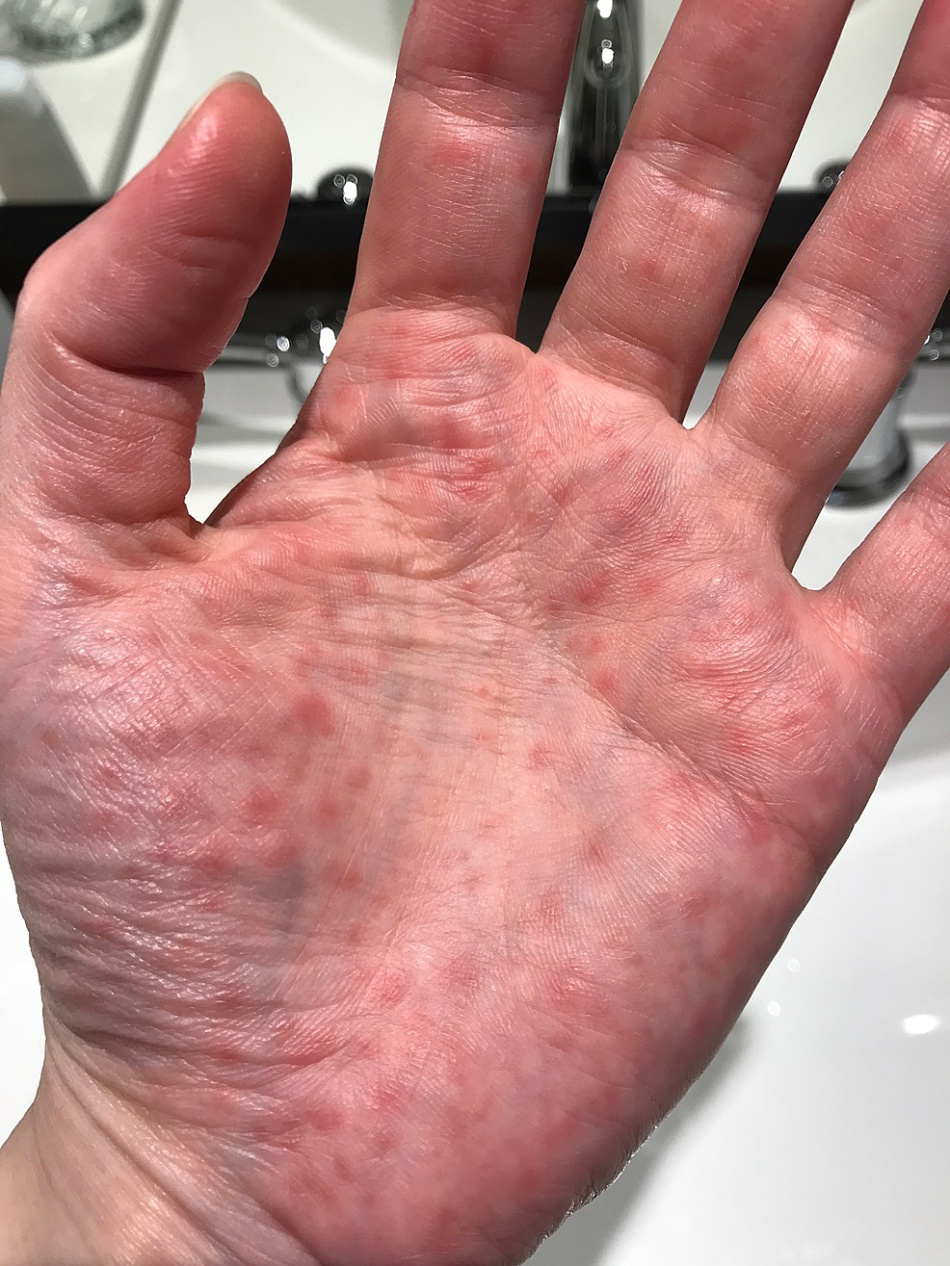
Pernio/chilblain lesions on the palm of patient’s hand

A 46-year-old lady with a past medical history of asthma, allergic rhinitis, chronic bronchitis, gastroesophageal reflux disease (GERD) and endometriosis presented to the allergy and immunology clinic for the evaluation of allergic rhinitis. She was experiencing rhinorrhea and bilateral eye discharge, with no symptoms of asthma, chronic bronchitis or GERD. She admitted to having recurring skin lesions on the palm of both of her hands and forearms since 2003, which was provisionally diagnosed as eczema and was treated unsuccessfully with halobetasol propionate 0.05% cream and triamcinolone acetonide 0.5% cream (Figure 1). She complained of erythematous, pruritic, burning, and painful papules on her hands, with eventual sloughing of the skin within 24 hours following cold exposure and resolution within three weeks. The patient stated that the only creams that relieved her symptoms were over-the-counter Aveeno Eczema Therapy and O'Keeffe's Working Hands. Her skin condition has remained stable and she came to the clinic for a second opinion. The patient currently lives in Michigan and has resided there for many years, with her symptoms appearing during the winter months. The patient stated that she had previously tried using gloves for three months in 2018 and that they only prevented smearing of the cream on her hands, rather than providing any improvement in her condition. She was evaluated by numerous doctors and dermatologists and she was diagnosed with pernio after ruling out other diseases such as Raynaud’s and lupus through negative blood tests and negative antinuclear antibody (ANA) and complement levels. She has not been ruled out for other malignancies or multiple myeloma. Her laboratory workup, including a complete blood count (CBC), complement levels, ANA, double-stranded DNA (dsDNA) and rheumatoid factor levels were all within normal limits. The patient also had no signs of a malar rash, fatigue, fever, weight loss, arthritis, or cardiac or renal issues. The patient had a significant family history of a maternal aunt diagnosed with lupus and her maternal grandfather had skin cancer. She never had COVID-19, denied any smoking and only indulges in alcohol on social occasions. This patient was interested in further ways she could manage her symptoms, as her usual medication of topical corticosteroids did not relieve her pain. Current management of her symptoms include preventing cold exposure by wearing gloves, using pocket warmers, continuing with over-the-counter creams and trying nifedipine, a calcium-channel blocker.

## Discussion

There is an unclear explanation for the pathogenesis behind pernio; however, studies have suspected it to be from an abnormal vascular response to cold exposure [2]. This cold-induced vasoconstriction or vasospasm causes hypoxemia that stimulates an inflammatory response. There have been case reports demonstrating that pernio may be a cutaneous manifestation of celiac disease, which this patient did not suffer from [2]. Histological clues that favor idiopathic pernio include keratinocyte necrosis, severe dermal edema, and dermoscopic white dots and lines [3]. Pernio is a disease of clinical diagnosis and skin biopsies are not necessary, unless a physician suspects a different disorder, as it can occur in patients with hematologic disorders, such as paraproteinemia, Raynaud’s, systemic lupus erythematosus, viral hepatitis or malignancy, and must be ruled out before suspecting pernio [4]. This patient tested negative for the aforementioned diseases. The major criteria to diagnose pernio are localized erythema and swelling involving the acral sites persisting for >24 hours [4]. The minor criteria include onset/worsening in the cooler months (November-March), histopathologic findings on skin biopsy (dermal edema with superficial and deep perivascular lymphocytic infiltrate), without findings of lupus erythematous, and a response to conservative treatments [4]. A diagnosis of pernio requires the major criteria and at least one of the three minor criteria [4]. This patient met the criteria for diagnosing pernio as she had the major criteria as well as a response to conservative treatment without findings of lupus erythematous.

Tests ordered when working up pernio include a CBC, serum protein electrophoresis, complement levels, antiphospholipid antibodies, and ANA levels [4]. Treatment for pernio includes preventing cold exposure as well as nifedipine, corticosteroid cream and pentoxifylline [1]. However, a recent clinical randomized, double-blind, placebo-controlled, cross-over trial found that nifedipine for the treatment of chronic chilblains is not as effective as a placebo and may cause harm, such as increased rates of peripheral edema [1]. Recently, this patient unsuccessfully tried corticosteroid cream; however, future treatments should be geared towards nifedipine and pentoxifylline. Also, the placebo effect seems to show promising results without any adverse effects and should also be considered.

There have also been newly released studies, showing that there may be a possible connection between the SARS-CoV-2 and pernio. Docampo-Simón et al. identified 88 patients with pernio-like lesions; however, a SARS-CoV-2 reverse transcription polymerase chain reaction (RT-PCR) was only positive in 14.8% of the cases, concluding no causal relationship [6]. This patient stated that she had never tested positive for COVID-19 and her symptoms began prior to the COVID-19 pandemic.

## Conclusions

In conclusion, pernio or chilblain disease is a clinical diagnosis that must be ruled out from other skin disorders such as Raynaud’s or acrocyanosis. Treatment ranges from cold exposure prevention to corticosteroids and nifedipine with varying clinical effectiveness. During the recent 2020-2021 COVID-19 pandemic, there have been numerous studies focusing on the relationship between SARS-CoV-2-associated pernio, as a possible late manifestation of COVID-19; however, there hasn’t been any direct causal link established. This clinical presentation highlights the importance of a physical examination and obtaining relevant medical history from the patient. After ruling out other diseases on the differential list, such as acrocyanosis, vasculitis and lupus, other clinical conditions should be considered. As pernio is a clinical diagnosis with a poorly understood pathology and mechanism, many patients may go undiagnosed for an extended period of time. It is essential to remember when a patient presents with pernio-like lesions and has negative test results, to consider alternative diagnoses and management strategies, including the placebo effect.
